# Cerebellar long-term depression and auto-immune target of auto-antibodies: the concept of LTDpathies

**DOI:** 10.1186/s43556-020-00024-x

**Published:** 2021-01-10

**Authors:** Hiroshi Mitoma, Jerome Honnorat, Kazuhiko Yamaguchi, Mario Manto

**Affiliations:** 1 Department of Medical Education, Tokyo Medical University, Tokyo, Japan; 2 French Reference Center on Paraneoplastic Neurological Syndromes, Hospices Civils de Lyon, Hôpital Neurologique, 69677 Bron, France; 3 Institut NeuroMyoGene INSERM U1217/CNRS UMR 5310, Université de Lyon, Université Claude Bernard Lyon 1, 69372 Lyon, France; 4 Department of Ultrastructural Research, National Institute of Neuroscience, National Center of Neurology and Psychiatry, Tokyo, Japan; 5 Unité des Ataxies Cérébelleuses, Service de Neurologie, Médiathèque Jean Jacquy, CHU-Charleroi, 6000 Charleroi, Belgium; 6 Service des Neurosciences, University of Mons, 7000 Mons, Belgium

**Keywords:** Cerebellar ataxias, Immune-mediated cerebellar ataxias, Long-term depression, Anti-mGluR antibody, Anti-VGCC antibody, Anti-GluR delta antibody

## Abstract

There is general agreement that auto-antibodies against ion channels and synaptic machinery proteins can induce limbic encephalitis. In immune-mediated cerebellar ataxias (IMCAs), various synaptic proteins, such as GAD65, voltage-gated Ca channel (VGCC), metabotropic glutamate receptor type 1 (mGluR1), and glutamate receptor delta (GluR delta) are auto-immune targets. Among them, the pathophysiological mechanisms underlying anti-VGCC, anti-mGluR1, and anti-GluR delta antibodies remain unclear. Despite divergent auto-immune and clinical profiles, these subtypes show common clinical features of good prognosis with no or mild cerebellar atrophy in non-paraneoplastic syndrome. The favorable prognosis reflects functional cerebellar disorders without neuronal death. Interestingly, these autoantigens are all involved in molecular cascades for induction of long-term depression (LTD) of synaptic transmissions between parallel fibers (PFs) and Purkinje cells (PCs), a crucial mechanism of synaptic plasticity in the cerebellum. We suggest that anti-VGCC, anti-mGluR1, and anti-GluR delta Abs-associated cerebellar ataxias share one common pathophysiological mechanism: a deregulation in PF-PC LTD, which results in impairment of restoration or maintenance of the internal model and triggers cerebellar ataxias. The novel concept of LTDpathies could lead to improvements in clinical management and treatment of cerebellar patients who show these antibodies.

## Introduction

During the last two decades, experimental and clinical studies have established the pathological roles of auto-antibodies against ion channels and synaptic receptors in limbic encephalitis [1–5]. Although auto-antibodies that target ion channels and synaptic machineries have been documented also in immune-mediated cerebellar ataxias (IMCAs), the types of auto-antibodies involved in IMCAs are different from those observed in limbic auto-immune encephalitis [6]. Anti-glutamate receptors, anti-GABA receptors and anti- leucine-rich glioma-inactivated 1(LGI1) antibodies (Abs) are rarely observed in IMCAs, whereas the association of CAs with anti-GAD65, anti-voltage-gated Ca channel (VGCC), anti-metabotropic glutamate receptor type 1 (mGluR1), and anti-glutamic receptor delta (GluR delta) Abs has been documented [7–12]. Especially, auto-antibodies against VGCC, mGluR and GluR delta are characteristically found in IMCAs, but not in auto-immune limbic encephalitis [6, 13]. These target molecules are involved in molecular cascades that induce long-term synaptic depression (LTD) of synaptic transmissions between parallel fibers (PFs) and Purkinje cells (PCs), a crucial form of synaptic plasticity in the cerebellum [6, 13].

In this review, we dissect the pathophysiological mechanisms underlying anti-VGCC, anti-mGluR1, and anti-GluR delta Abs-associated cerebellar ataxias (CAs), and address pathophysiological roles of impaired PF-PC LTD. Thus, we discuss (*1*) the roles of these auto-immune target molecules (VGCC, mGluR1 and GluR delta) in the induction of LTD, and (*2*) the clinical profiles of the IMCAs subtypes associated with these auto-antibodies. We argue that PF-PC LTD dysfunction is one of the final common pathophysiological mechanisms in these three subtypes of IMCAs. Auto-immune response might impair the restoration or maintenance of the internal model, resulting in the development of CAs.

## Physiology of long-term depression between parallel fibers and Purkinje cells

### Neuronal circuit of cerebellar cortex and PF-PC LTD

**Fig. 1:**
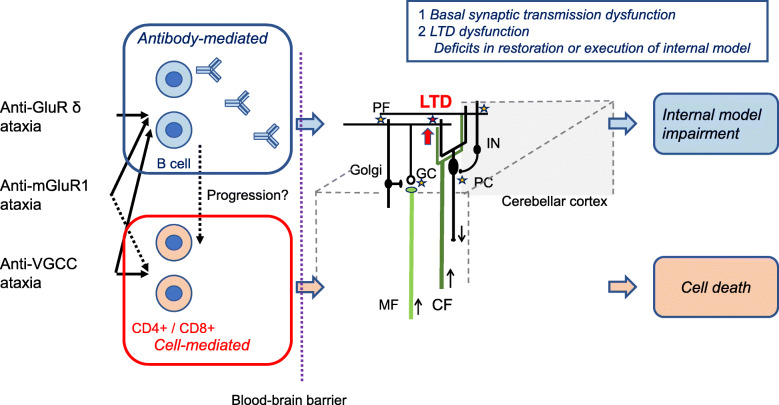
Schematic diagram of the pathophysiological mechanisms underlying anti-VGCC, anti-mGluR1, and anti-GluR delta-associated cerebellar ataxias. The antibody-mediated mechanisms include dysfunction of basal synaptic transmissions and long-term depression (LTD), leading to impairment of the internal model. These functional disorders are followed by cell-mediated cell death, depending on the auto-immune stimulus. PC; Purkinje cell, GC; granule cell, IN; inhibitory interneurons, CF; climbing fiber, PF; parallel fiber, MF; mossy fiber

The cerebellar cortex receives two excitatory glutamatergic input fibers, namely, mossy fibers (MFs) and climbing fibers (CFs) (Fig. 1). MFs make synaptic connections with granule cells (GrCs) and cerebellar nucleus (CN) neurons. PFs, axons of GrCs, meet with multiple PCs, and form a glutamatergic synapse with a single PC [14, 15]. At the PF-boutons, action potentials activate VGCC (mainly P/Q-type VGCC) [16]. Glutamate, released from the PF terminals in a Ca^2+^-dependent manner, activates AMPA-type Glu-receptors at the dendritic spine of PC. The PF-PC synaptic input generates and modulates simple spikes, whose firing pattern is affected by voltage-gated K channels and Ca^2+^-dependent K channels [17–19]. The PF-PC synapse is bound by a set of synaptic adhesion molecules, i.e., neurexin-cerebellin1-GluR delta [20, 21]. By contrast, a single CF that originates from the inferior olive nucleus establishes strong multisite synapses on the dendrites of a single PC [14, 15].

Simultaneous and repetitive activation of PF and CF depresses PF-excitatory postsynaptic currents with a long-term time course, which is termed the LTD [15, 22]. Based on the finding that CF fire at high probability in case of motor failures [23], it has been proposed that CF carry an error signal of motor performance and PF-PC LTD provides the mechanism for motor learning [15], though this is still debated.

### Molecular mechanisms underlying PF-PC LTD

**Fig. 2:**
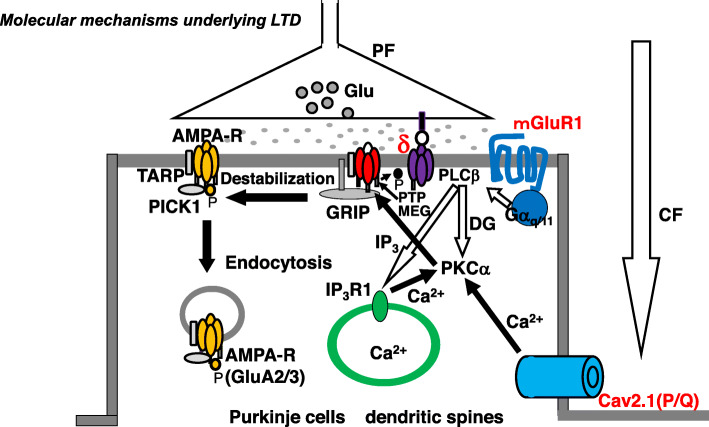
Schematic diagram of long-term depression (LTD) at excitatory synapses between parallel fibers and Purkinje cells. The climbing fiber input elicits complex spikes through the activation of dendritic P/Q type Ca^2+^ channels, leading to an increase in intracellular calcium concentration ([Ca^2+^]_i_). On the other hand, the parallel fiber input activates metabotropic glutamate receptor-PLCβ-IP_3_ signaling pathways, resulting in an increase in [Ca^2+^]_i._ The conjunctive activation of these two pathways increases [Ca^2+^]_i_ more than the additive level. The high [Ca^2+^]_i_ activates PKCα, and PKCα phosphorylates GluA2 of the AMPA (α-Amino-3-hydroxy-5-methyl-4-isoxazolepropionic acid) receptor, which results in detachment of the AMPA receptor from scaffold proteins and its internalization with PICK1 in an AP2 and clathrin-dependent manner. CF; climbing fibers, PF; parallel fibers, Glu; glutamate; AMAPA-R; AMPA receptor, mGluR1; metabotropic glutamate receptor, Cav2.1 (P/Q); P/Q type Ca^2+^ voltage-gated channel, PLC; phospholipase C, PKC; protein kinase C, IP_3_; Inositol triphosphate, GRIP; Glutamate receptor interactive protein, TARP; transmembrane AMPA receptor regulatory proteins, PICK1; protein interacting with C kinase, δ; GluR delta 2, PTPMEG; megakaryocyte protein phosphatase

Conjunctive stimulation of CF and PF causes LTD of PF-PC synaptic transmissions both in vivo [22] and in vitro [24–26]. CF input elicits an increase in [Ca^2+^]_i_ through the VGCC (P/Q-type) (Fig. 2) [27]. PF inputs in dendritic spines activate the mGluR-PLCβ-IP_3_ signaling pathways, which elicits Ca^2+^ release from the Ca^2+^-stores in the endoplasmic reticulum (ER) through IP_3_ receptors and, consequently, increases [Ca^2+^]_i_ (Fig. 2) [28].

Simultaneous activation of VGCC (P/Q-type) and mGluR1 elicits a series of events, including an increase in [Ca^2+^]_i_ to levels higher than the additive level [29], which leads to the activation of PKCα, which in turn phosphorylates GluA2-C terminus, ultimately leading to the detachment of AMPA receptors, including phosphorylated GluA2, from the scaffold protein and its internalization with PICK1 in AP2- and clathrin-dependent manners (Fig. 2) [30]. Also CaMKII [31] and NMDA-receptor [32, 33] are involved in PF-PC LTD induction.

GluR delta, a synaptic adhesion molecule specific to the PF-PC synapse [21], is also involved in LTD. Antibodies against the N-terminus region (H2 ligand binding site) decrease the amplitude of evoked excitatory postsynaptic currents [34], possibly by suppressing the PF-PC synaptic interaction. In addition, the antibodies suppressed induction of LTD in culture preparations. Suppression of GluR delta expression in cultured PCs by antisense oligonucleotides also resulted in severe impairment of LTD [35]. These findings highlight the importance of GluR delta in LTD, for reasons other than maintenance of synaptic interaction. The cytoplasmic terminus of GluR delta binds to megakaryocyte protein phosphatase (PTPMEG), which dephosphorylates tyrosine 876 in GluA2. Dephosphorylation of this site is necessary for PKCα-induced phosphorylation of serine 880, an essential step in the internalization of AMPA receptors [36]. Thus, GluR delta is assumed to gate PF-PC LTD by coordinating the interaction between the two phosphorylation sites in GluA2 [36]. However, whether this dephosphorylation through GluR delta-PTPMEG interaction is impaired by antibodies against GluR delta extracellular region is unknown.

In conclusion, an increase in [Ca^2+^]_i_ is triggered by conjunctive activation of VGCC and mGluR1, and the subsequent AP2-and clathrin-dependent endocytosis of AMPA receptors is an essential process in PF-PC LTD. Furthermore, endocytosis is gated by dephosphorylation of T876 of the GluA2-C terminus through GluR delta-PTPMEG interaction.

### Internal model and PF-PC LTD

#### Internal model and cerebellar ataxias

Evidence suggest the presence of internal models embedded in the cerebellum to elaborate coordination of motor and cognitive commands [37, 38]. The internal model, that emulates the dynamics of body and environments, are essential for integrating the movements of the body parts without the need for sensory feedback [39]. An internal inverse model transforms the desired trajectory into motor commands [40, 41] and is involved mainly in cerebellar ocular motor controls [42]. On the other hand, an internal forward model computes the future state of the body based on the current estimate of the body and efferent signals of motor control [43–45]. Evidence suggests that online predictive computations that employ the internal forward model coordinate limb voluntary movements [46–50]. The predictive computation of the forward model affords coordination of multiple degrees of freedom and appropriate timing of muscle activities [39]. Since the deficits in predictive activation of the triceps muscles results in dysmetria, it is assumed that dysmetria occurs as a result of impaired predictive computation of the internal forward model in the cerebellum [39]. Other ataxic symptoms including adiadochokinesis and ataxic gaits could be attributed to impairments of the internal forward model [39].

#### Internal model and synaptic plasticity

Through cerebellar leaning, the internal model is assumed to be acquired and continuously updated following changes in the external factors. To execute the task of reaching, for example, the cerebellum organizes compound movements based on the physical properties of various body parts, including muscle strength, segment inertia, joint viscosity, and segmental interaction, which are stored in the cerebellum [51].

In the cerebellar cortex, multiple forms of synaptic plasticity at different sites are induced during procedural memory formation (Fig. 2) [52]. At the cerebellar input synapses (MF-GrC), high-frequency bursts (> 250 msec) of MF can induce MF–GrC LTP, whereas low-frequency bursts induce LTD [53, 54]. Repetitive stimulation of PF alone causes potentiation of PF-PC EPSP [25, 55]. This postsynaptic type of LTP can reset PF-PC LTD, and vice versa; PF-PC LTD can reset PF-PC LTP, thus PF-PC LTP and PF-PC LTD mutually counterbalance each other [56–59]. As for the inhibitory input to PC, GABA-mediated inhibitory synaptic transmission undergoes a long-lasting rebound potentiation (RP) after activation of excitatory CF inputs [60]. Thus, the multiple forms of synaptic plasticity in the cerebellar cortex challenges the concept of the critical role of LTD in cerebellar motor learning. Furthermore, in certain mutant mice that lack PF-PC LTD, show normal motor learning. Based on such conclusion, some researchers have argued that LTD is not essential for motor learning [61, 62].

Recent experiments have provided substantial evidence for the essential role of PF-PC LTD in motor learning. First, interpretation of the results of gene-manipulation experiments should be assessed carefully. In general, compensatory mechanisms of synaptic plasticity are probably expressed in gene-manipulated animals, and the conditions necessary to induce compensated synaptic plasticity could be different from the ordinal experimental conditions that induce PF-PC LTP or LTD in the wild type animal. Actually, several types of PF-PC LTD-inducing stimulation protocols have been used to induce LTD in the same mutant mouse [63], indicating that LTD-hypothesis could not be ruled out. Second, the use of a new optogenetic blocker of endocytosis allows reversible PF-PC LTD blockade. This tool (PhotonSABER) enables the temporal, spatial, and cell-type specific control of AMPA receptor endocytosis at active synapses. Blockade of PF-PC LTD by photostimulation in vivo resulted in impairment of vestibulo-ocular reflex (VOR)-adaptation [64]. This photoactivation of PhotonSABER neither affected RP nor PF-PC LTP. Although RP and PF-PC LTP were intact, VOR-adaptation was impaired following blockade of PF-PC LTD, thus suggesting the importance of LTD in cerebellar motor learning.

In conclusion, CAs can be attributed to impaired predictive computation of the internal forward model in the cerebellum. The internal model is formed and continuously updated by cerebellar learning processes. Motor learning depends on various forms of synaptic plasticity in the cerebral cortex through an increase in the contrast of input signals, efficiency of learning, and accuracy of learned behavior. However, PF-PC LTD seems to play a crucial role in motor learning by adjusting the final stage of the cerebellar cortical integration.

## LTD-related synaptopathies in immune-mediated cerebellar ataxias

### Anti-VGCC antibody-associated cerebellar ataxia

#### Clinical profiles

Anti-VGCC antibodies were initially described in association with Lambert-Eaton myasthenia syndrome (LESM) [3]. However, the association of CAs with anti-VGCC Abs has also been described in patients with paraneoplastic cerebellar degeneration (PCD) with or without LEMS [65]. The most frequent associated cancer is small cell lung cancer (SCLC) [66]. Auto-antibodies against the P/Q-type VGCC, the main antigen, was detected in 78% of patients with PCDs and LEMS, and in 20% of anti-Hu Ab-negative patients with PCDs without LEMS [66]. The patients showed subacute pancerebellar ataxias, and there were no differences in the clinical profiles of PCD patients with and without anti-VGCC Ab [67]. Anti-VGCC Abs were also identified in patients with non-paraneoplastic IMCAs [65] and a large-scale study showed that anti-VGCC Abs were positive in 8 of 67 patients who showed chronic cerebellar degeneration [68].

The therapeutic response depends on the background. A study on 16 anti-VGCC Ab-positive patients with PCD and SCLC found poor benefits to immunotherapies [67]. Of these patients, one showed complete recovery, five showed stabilization at a low Rankin score, and five stabilized or worsened at high Rankin scores. The median survival time of these patients was 12 months. On the other hand, good prognosis was reported in patients with non-paraneoplastic conditions [68]. Immunotherapies have been used in patients with paraneoplastic and non-paraneoplastic conditions, and these included intravenous immunoglobulins (IVIg), prednisone, and mycophenolate mofetil (Table 1).

**Table 1:** Clinical profiles of anti-VGCC, anti-mGluR and GluR delta antibodies-associated cerebellar ataxias

	Anti-VGCC	Anti-mGluR1	Anti-GluR delta
Prevalence in IMCAs	Sometimes	Rare	Rare
Trigger of autoimmunity	Mainly with paraneoplasia (SCLS, prostate adenocarcinoma, non-Hodgkin’s lymphoma). A few without paraneoplasia	Some with paraneoplasia (Hodgkin’s lymphoma, prostate adenocarcinoma). Others without paraneoplasia and infection	Infection, vaccination
Age, gender	50–60s	Median 55 years (IQR 43–64), 43% females	Children
Features of CAs	Pancerebellar ataxias	Gait and limb ataxias	Gait ataxia associated with limb ataxia and dysarthria
MRI^a^		Variable: From no to mild atrophy	No atrophy
Outcomes	Paraneoplasia: Variable. From good to poor response to IVIg, prednisone and mycophenolate mofetil. Non-paraneoplasia: Improvement reported.	Paraneoplasia: Variable. From good response to poor response to IVIg, PE.. Non-paraneoplasia: Generally good response to IVIg, steroid, mycophenolate, and rituximab.	Generally good response to IVIg or IVMP.
	Paraneoplasia, *n* = 11 [64]	Paraneoplasia /Non-paraneoplasia [69]	Non-paraneoplasia, *n* = 3 [70–72]
Full or partial recovery	1 (10%)	10 (40%)	2 (67%)
Stabilization	6 (55%)	14 (56%)	1 (33%)
Persistent aggravation	5 (45%)	1 (4%)	0

*IMCAs* Immune-mediated cerebellar ataxias, *SCLS* Small cell lung cancer, *IVIg* Intravenous immunoglobulins, *IVMP* Intravenous methylprednisolone, *PE* Plasma exchange

Interpretation: the occurrence of cerebellar atrophy appears variable from case to case. The mechanisms of the atrophy remain to be discovered. This occurs also in other immune-mediated cerebellar ataxias

#### Physiological actions of antibodies

A polyclonal peptide Ab against the major immunogenic region in P/Q-type VGCCs (the extracellular domain-III S5–6 loop) impaired the functions of neuronal and recombinant P/Q-type VGCC, and elicited a decrease in Ca^2+^ currents, leading to impaired synaptic transmission between PF and PC [73]. A reduction in P/Q-type VGCC was also observed in the autopsies of three patients with PCDs and LEMS [74]. In experimental studies, ataxic symptoms were induced in mice by intrathecal administration of serum IgGs obtained from anti-P/Q type VGCC Ab-positive patients with PCDs and LEMS [75]. However, the actions of anti-VGCC Ab on LTD have not been studied.

### Anti-mGluR1 antibody-associated cerebellar ataxia

#### Clinical profiles

The association of anti-mGluR1 Ab with CAs has been reported initially in two patients with Hodgkin’s lymphoma [76] and one patient with prostate adenocarcinoma [69]. The response to immunotherapy varied among the three patients; the two patients with Hodgkin’s lymphoma responded well to the combination of plasma exchange, IVIg and oral prednisone, whereas the other patient with prostate cancer showed no objective improvement after plasma exchange.

On the other hand, the association of anti-mGluR Ab with CAs was also described in non-paraneoplastic conditions [70, 77]. The clinical course is now better known for portraying a series of 11 new patients and 19 previously reported patients (Table 1) [71]. The main neurological manifestations were subacute cerebellar gait and limb ataxias in 25 of these 30 patients (86%), sometimes associated with extra-cerebellar symptoms, such as behavioral changes (irritability, apathy, mood, personality change, psychosis with hallucinations, and catatonia), cognitive changes (memory problems, executive functions and spatial orientation deficits) or dysgeusia. Seizures were uncommon. Three of the 26 patients (11%) had paraneoplastic conditions (cutaneous T lymphoma and Hodgkin’s lymphoma). Serological tests identified anti-mGluR1 Ab in both the serum and CSF, together with evidence of pleocytosis in the CSF. MRI showed normal in 12 of the 19 patients (63%) at the onset. Abnormal findings included T2/FLAIR hyperintensities or leptomeningeal gadolinium enhancement. At follow-up, MRI showed cerebellar atrophy in 10 of the 12 (83%). Twenty-five of the 30 (83%) patients received immunotherapies, including IVIg, steroids, mycophenolate mofetil, cyclophosphamide, and rituximab alone or in combinations. Ten patients (40%) showed significant improvements or complete resolution of symptoms, and 13 patients (52%) showed stabilization or mild improvement. Although two patients not associated with tumors died, one patient initialized improved and died due to unknown etiologies. Thus, only one patient showed progressive worsening of CAs.

#### Physiological actions of antibodies

IgGs purified from the sera of patients with Hodgkin’s lymphoma blocked glutamate-stimulated formation of inositol phosphates in mGluR1α-expressing Chinese-hamster-ovary cells [76]. The IgGs blocked the induction of LTD in tissue slices [72]. Application of IgGs in the subarachnoid space elicited ataxic gaits in mice, which disappeared after absorption of anti-mGluR1 Ab [75, 76], while administration of the same Ab in the flocculus also evoked acute disturbances in compensatory eye movements [72]. A recent study confirmed that CSF from the patients decreased mGluR1 clusters in cultured neurons [71].

### Anti-GluR delta antibody-associated cerebellar ataxia

#### Clinical profiles

The association of anti-GluR delta Ab with CAs has been described in a few patients with non-paraneoplastic conditions [78–81]. The conditions affected children aged 8 months to 13-years and medical history showed infection or vaccination preceding CAs. These patients exhibited prominent gait ataxia associated with variable degree of limb ataxia and dysarthria with an acute time-course. Serological tests were positive for anti-GluR delta Ab in the serum and CSF and laboratory tests showed pleocytosis without oligoclonal bands in the CSF, while MRI demonstrated no evidence of cerebellar atrophy. Most of these patients responded well to immunotherapy of IVIg or IVMP coupled with clinical improvement in CAs (Table 1). Another 25-month-old girl with chronic recurrent CA positive for anti-GluR delta Ab also showed a good response to corticosteroid therapy [82].

#### Physiological actions of antibodies

Although subarachnoidal injection of Ab against the H2 ligand binding site of GluR delta elicited the development of ataxic phenotype in mice and the Ab impaired simplified LTD in cultured PC [34], the actions of anti-GluR delta Ab on LTD in mature PC with intact physiological neuronal circuitry remain to be investigated in adult cerebellar slices.

## Possible pathophysiological mechanisms

### Diversity and composite in auto-immune mechanisms

Anti-VGCC, anti-mGluR1, and anti-GluR delta Abs-associated CAs show divergent clinical profiles, for example, different background (paraneoplastic or non-paraneoplastic) and age preponderance. In addition to the clinical diversity among these subtypes, compound and overlapped immune mechanisms appear to be involved even in each etiology, especially in anti-VGCC Ab-associated CA. Both in vitro and in vivo studies have shown that antibodies towards VGCC impaired the release of neurotransmitters, leading to the development of CAs. Despite this evidence of functional disorders, cerebellar cell loss was found in postmortem studies [74]. This discrepancy suggests that antibody-induced functional disorders are followed by additional cell-mediated mechanisms that lead to cell death. It is suggested that in paraneoplastic conditions, the auto-immune signal is augmented in a positive feed-back fashion [83]. For example, persistence of the auto-immune responses to cancer cells would continue to cause secretion of cytokines that elicit sustained vascular hyperexcitability and infiltration of immune cells. Death of cancer cells subsequently leads to lasting release of intracellular antigens. These persistent and amplified auto-immune conditions might recruit sequential responses, from antibody-mediated functional disorder to additional cytotoxic T-cell-mediated cell death [75].

In conclusion, various backgrounds (paraneoplastic, infectious or unknown conditions) can trigger auto-immune attacks against the cerebellar circuits in anti-VGCC, anti-mGluR1, and anti-GluR delta Abs-associated CAs, where multiple auto-immune reactions can be elicited depending on the quality, intensity or duration of the auto-immune stimulus. Sequential responses, from humoral functional disorders to cell-mediated irreversible cell death, might sometimes occur (Fig. 1).

### Functional disorders and internal model impairments

In spite of the diversity and composite of auto-immune mechanisms, anti-VGCC, anti-mGluR1, and anti-GluR delta Abs-associated CAs also appear to have a single final common pathway. Patients with these three subtypes commonly show good prognosis without or only mild cerebellar atrophy when associated with non-paraneoplastic conditions, which is contrast to the poor prognosis in anti-GAD ataxia. The anti-GAD ataxia sometimes shows resistance to immunotherapy, resulting in severe CAs associated with marked cerebellar atrophy and cell death [84]. Thus, the good therapeutic response in these three etiologies can be an outstanding feature of IMCAs, and reflects staying of functional disorders, dysfunction of the internal model. Taken together, we propose that anti-VGCC, anti-mGluR1, and anti-GluR delta Abs commonly distort the internal model leading to the development of cerebellar functional disorders.

### LTD as a common final pathophysiological pathway?

One possible mechanism for the functional disorders is deficit in the basal synaptic transmission in the cerebellar circuits. In addition to anti-VGCC Ab [73], impaired synaptic transmission on PC was documented in anti-mGluR1 Ab [85] and anti-GluR delta Ab [34]. Disturbance of the basal synaptic transmission subsequently lead to distortion of the internal model (Fig. 1).

The alternative mechanism is a deficit in PF-PC LTD, since VGCC and mGluR1 are molecules required for the increase in Ca^2+^ concentrations and subsequent induction of PF-PC LTD, while GluR delta plays an important role in AMPA receptor trafficking. Consistently, it has been demonstrated that IgGs from some of these patients impaired the induction of LTD [72]. The physiological relationship between LTD dysfunction and internal model impairment is as follows (Fig. 1).

(*1*) When these auto-antibodies interfere with the basal synaptic transmission, any impairment of the internal model should be compensated for by synaptic plasticity, including PF-PC LTD. Thus, PF-PC LTD dysfunction can distort internal model restoration.

(*2*) It has been a matter of debate whether PF-PC LTD is required to develop normal motor performance [86]. However, recent studies suggest that PF-PC might be involved in execution of the internal model. First, using the method of conditional knockout mice (a tetracycline-controlled gene expression system), acute blockade of mGluR impaired PF-PC LTD and simultaneously elicited motor incoordination without affecting basal synaptic transmission [85]. Second, a study on neural activities in monkeys indicates a facilitative role of PF-PC LTD in the online predictive controls [87]. The cerebellar output, before the movements, is generated by phasic suppression of PCs and concomitant activation of the dentate nucleus cells (*disinhibition mechanism*) [87]. Impairments in the disinhibition elicit a delay in movement initiation, which was termed asthenia by Holmes G [88]. Notably, the phasic suppression on PCs is assumed to be tuned by facilitation by PF-PC LTD [87].

It is uncertain whether dysfunction of LTD can directly alter the internal model. This hypothesis will be validated using in vitro and in vivo experiments in which PF-PC LTD is directly blocked by molecular manipulation of AMPA receptor endocytosis (e.g., PhotonSABER method [64] or manipulation of transmembrane AMPA receptor regulating proteins [89]). In these in vivo models, impairments in execution of the internal model will be examined by monitoring predictive muscle activations, an index for on line predictive controls [39]. The long-term alterations will be analyzed using in learning paradigms.

## Conclusion

Anti-VGCC, anti-mGluR1, and anti-GluR delta Abs-associated CAs show divergent clinical profiles in auto-immune background (paraneoplastic or non-paraneoplastic) and susceptible age. However, these patients commonly show good prognosis without cerebellar atrophy in non-paraneoplastic conditions, suggesting functional disorders in the internal model. These autoantibodies would impair basal synaptic transmissions in the cerebellar cortex. Besides, the dysregulated synaptic plasticity-related dysfunction might be overlapped, since VGCC, mGluR1, and GluR delta are involved in the induction of PF-PC LTD. Deficits in restoration of the internal model might occur. Furthermore, dysregulated PF-PC LTD might impair the execution processes of the internal model. In order to highlight the synaptic plasticity-related dysfunction, we propose the concept of “LTDpathies” to gather cerebellar ataxias associated with antibodies targeting the critical mechanism of PF-PC LTD. Auto-antibodies impair fundamental paths of a key-plasticity in the cerebellar cortex. “LTDpathies” share a favorable prognosis, highlighting that this group of disorders does not target the cerebellar reserve in terms of neuronal destruction, but rather blocks adaptative mechanism in the cerebellum.

## Data Availability

The concept reported in this manuscript is not associated with raw data.

## Abbreviations

Ab: Antibody
CFs: Climbing fibers
CN: Cerebellar nucleus
GluR delta: Glutamic receptor delta
GrCs: Granule cells
IMCAs: Immune-mediated cerebellar ataxias
IVIg: Intravenous immunoglobulins
LESM: Lambert-Eaton myasthenia syndrome
LGI1: Leucine-rich glioma-inactivated 1
LTD: Long-term synaptic depression
MFs: Mossy fibers
mGluR1: Metabotropic glutamate receptor type 1
PCD: Paraneoplastic cerebellar degeneration
PCs: Purkinje cells
PFs: Parallel fibers
PTPMEG: Megakaryocyte protein phosphatase
RP: Rebound potentiation
SCLC: Small cell lung cancer
VGCC: Voltage-gated Ca channel
VOR: Vestibulo-ocular reflex
